# Impact of Lewy bodies disease on visual skills and memory abilities: from prodromal stages to dementia

**DOI:** 10.3389/fpsyt.2024.1461620

**Published:** 2024-12-10

**Authors:** Cinzia Bussè, Micaela Mitolo, Stefano Mozzetta, Annalena Venneri, Annachiara Cagnin

**Affiliations:** ^1^ Department of Neuroscience, University of Padua, Padua, Italy; ^2^ Padova Neuroscience Center, University of Padova, Padua, Italy; ^3^ Department of Medicine and Surgery, University of Parma, Parma, Italy; ^4^ IRCCS Istituto delle Scienze Neurologiche di Bologna, Programma Neuroimmagini Funzionali e Molecolari, Bologna, Italy; ^5^ Department of Life Sciences, College of Health, Medicine, and Life Sciences, Brunel University London, London, United Kingdom

**Keywords:** Lewy bodies disease, dementia, mild cognitive impairment, visual impairment, memory, cognition

## Abstract

Dementia with Lewy bodies (DLB) and its prodromal presentation with mild cognitive impairment is characterized by prominent deficits in attention/executive domains and in visual processing abilities with relative sparing of memory. Neuropsychological research is continuously refining the tools to define more in detail the patterns of relatively preserved and impaired cognitive abilities that help differential diagnosis between DLB and Alzheimer disease (AD). This review summarizes the main studies exploring specific cognitive tasks investigating different visual processing abilities and verbal memory that better differentiate DLB from AD. The findings provide evidence that substantial impairments in visual-spatial and visual-constructional abilities and relatively better performance on memory tasks that depend on hippocampal function characterize the prodromal stage of DLB. The ability to detect early indicators of prodromal DLB through clinical and cognitive assessments is the first step to guide instrumental diagnostic work-ups and provide the opportunity for early intervention.

## Introduction

The diagnosis of dementia with Lewy bodies (DLB) requires the presence of cognitive impairments with prominent and early deficits of attention, executive functions, and visuoperceptual abilities and at least one of the core clinical features among fluctuation of cognition, recurrent visual hallucinations, and spontaneous extrapyramidal signs (1). Memory impairment is relatively less severe early in the course of the disease, with a preservation of the ability to consolidate information (2). New consensus criteria for the diagnosis of DLB published in 2017 (3) increase diagnostic sensitivity and specificity by including detection of Rem Sleep Behavior disorder (RBD) as the core clinical feature and adding diagnostic value to investigations detecting nigrostriatal dysfunction with DaT scan SPECT, cardiac sympathetic denervation with MIBG SPECT, or RBD with polysomnography. As for the cognitive profile, the new criteria maintain the same framework than the previous set of criteria.

The need to improve sensitivity of clinical diagnosis of DLB comes from the high frequency of DLB cases missed or misdiagnosed as AD in clinical settings (4). It is recognized that patients with DLB usually display a different pattern of cognitive decline from that detected in AD, with worse performance in attentional and executive tasks (5, 6) and in visuospatial/constructional and visuoperceptual abilities (6–13) relative preservation of verbal episodic memory at least in the early stages of DLB. Thus, the combination of worse visual abilities (visual attention, visuoconstructional, spatial and perceptual abilities) and a better performance on delayed memory recall may offer the best discrimination between DLB and AD at least in the early/moderate stages.

Later, Mc Keith and colleagues (2020) (14) published a set of new research criteria for the diagnosis of Lewy bodies disease (LBD) as prodrome of DLB, recognizing at least three possible presentations with cognitive deficits, psychiatric manifestations and delirium. The most easily recognized form of prodromal DLB is that presenting with Mild Cognitive Impairment (MCI). Essential for a diagnosis of MCI with Lewy bodies (MCI-LB) is the presence of a cognitive complaint from the patient or from an informant or clinician who knows them and has observed a decline. Also, the presence of deficits in one or more cognitive domains is required, usually within visuospatial or attentive-executive functions, with a severity greater than that expected in normal aging. Moreover, although people with MCI may be less efficient, their cognitive deficits should not be severe enough to interfere with their typical daily functioning (14).

In this article the most salient literature exploring the diagnostic accuracy of testing visual abilities and memory in the differential diagnosis with AD has been reviewed with a focus on changes in cognitive performance across the LBD continuum from the prodromal stage to conversion to dementia.

## Visual abilities in dementia with Lewy bodies

The presence of visuospatial/constructional deficits at their first assessment is very common in autopsy-confirmed DLB patients (12). In this autopsy-based study, the best model to predict the diagnosis of DLB was the presence of visual hallucinations (positive predictive value: 83%), while lack of visuospatial deficits represented a highly negative predictor of DLB (negative predictive value: 83%).

Different tools are available to assess visual abilities, from simple screening instruments to more complex neuropsychological batteries that could disentangle the visuoconstructional, visuoperceptual and visuospatial components of tasks.

Ala and colleagues in 2001 used a simple tool as the copy of intersecting pentagons from the MMSE to compare the visuoconstructional abilities of patients with pathologically confirmed diagnosis of DLB and AD (8). Drawings of intersecting pentagons were analyzed as correct/incorrect. At comparable levels of severity, patients with DLB were more likely to draw an incorrect pentagon than those with AD with a sensitivity of 88% and a specificity of 59% in identifying DLB. Consistent with this finding, another study reported that impairment in copying the MMSE pentagons was associated with deficits of visuoperception and praxis in DLB, while in AD it was related to a more global cognitive impairment (10).

A qualitative scoring method for the pentagons copy test (QSPT) of the MMSE was proposed and applied to patients with mild-to-moderate DLB (mean MMSE score =19/30) (15). The DLB group performed worse than the AD group on a variety of parameters of the qualitative scoring method including the number of angles, distance/intersection, closure/opening, rotation, while no significant differences between the two groups were found on the closing-in phenomenon (mean QSPT total score: DLB: 6.74 ± 4.40, AD: 9.35 ± 3.41; p= 0.002).

The QSPT of the MMSE was also retrospectively applied to a cohort of DLB patients whose diagnosis had been subsequently confirmed neuropathologically, demonstrating that an alteration of the number of angles of the intersecting-pentagons was present in 33.3% of patients with DLB at the time of their first evaluation (mean MMSE score: 24.5/30), compared with 6.3% of AD patients (mean MMSE score AD: 24.8/30) (16).

It is, therefore, well established that DLB patients are usually more impaired in copying tasks (cube or intersecting-pentagons copy) than patients with AD, while both groups tend to be equally impaired in drawing a figure from memory (17–19) Some studies have compared the sensitivity of copying tasks with other visuoconstructional tasks such as the clock drawing test. In a study by Yamamoto using the Montreal Cognitive Assessment (MoCA) in 73 DLB and 57 AD patients in their mild-to-moderate stage of the disease, the cube-copy performance was similar between DLB and AD while the score obtained on the clock drawing test was worse for DLB (20).

Other studies have demonstrated that performance on the copy of complex figures, such as the Rey-Osterrieth Complex Figure, was similarly impaired in DLB and AD in both moderate stage (MMSE=20/30) of the disease (18) or early/mild stage (mean MMSE=24/30) (19). However, a qualitative scoring of different aspects of the Rey Complex Figure could capture differences in planning strategies in visuoconstructional tasks that could help detection of DLB patients more easily than using the total score.

Noe et al. (17) investigated copy abilities using 5 visual designs from the Benton Visual Retention task (21) and the Rosen Drawing test (22) in DLB, Parkinson-dementia and AD. The findings showed that patients with DLB and Parkinson-dementia (PDD) performed significantly worse than those with AD, showing more severe visuospatial and constructional deficits than AD patients (17).

The Visual and Object Space Perception battery (VOSP) (23) offers the possibility to disentangle visuoperceptual difficulties from constructional or spatial deficits on a set of different tasks that emphasize the bottom-up aspects of visual cognition. Calderon et al. was the first to apply this cognitive instrument to DLB and AD patients showing more severe impairments in early DLB on tests assessing both visuoperceptual (Fragmented Letters and Object Decision items) and visuoconstructional (Cube Analysis item) functions (24). Impairments of this type typically occur at a later stage in AD.

Similar results were found by Mosimann et al. using different sets of object and form perception tasks (length, size, angle) targeting the visual ventral stream and dot position and motion perception tasks (dot position, motion perception, visual counting) targeting the dorsal visual stream, in DLB, PDD and AD patients as well as matched healthy subjects. DLB and PDD patients were more impaired in all tasks than patients with AD. Moreover, DLB performed worse on object perception than space/motion perception tasks (11).

Furthermore, Mori et al. used a battery of 4 test items to examine various aspects of visual abilities in patients: (1) the discrimination of object size task assessed elementary visual perception, (2) the form discrimination task investigated complex visuoperceptual function of 2-dimensional visual stimuli, (3) the overlapping figures identification task assessed the abilities to detect concrete shapes and recognize objects, and (4) the visual counting task was used to examine the ability to explore and identify the spatial relationship among visual stimuli by counting targets (7). The results of this study showed that patients with probable DLB scored significantly lower than patients with AD of similar moderate severity on all visual tasks, indicating that both higher-order visual cognitive functions and elementary visual perception abilities are defective in DLB. As the disease progresses, the cognitive profiles of DLB and AD become similarly impaired. They speculated that early dysfunction of specific brain regions in DLB may hamper performance on these tests, namely: the occipital visual cortex for basic perception as required by task 1, the occipitotemporal association cortex for form discrimination (tasks 2 and 3) and the occipitoparietal cortex for visual counting (task 4).

In summary, although some differences emerge among studies probably related to the heterogeneity of cognitive instruments applied at different levels of disease severity, a clear cognitive profile of impairments involving visuoconstructional and visuoperceptual abilities more severely in DLB than in AD is detectable in the earliest stages of disease, while with disease progression visuoperception abilities may be similarly impaired.

## Visual abilities in the prodromal stage of dementia with Lewy bodies

MCI is conceived as a state intermediate between normal cognitive function and dementia (25). DLB can be preceded by amnestic or non-amnestic MCI, although cases involving non-memory domains, such as attention/executive, visuospatial or language, are more likely to progress to DLB than single-domain amnestic MCI (26–28).

Research on MCI-LB, i.e. the earliest stages of DLB without dementia, is relatively recent. It has been recently proposed that a diagnosis of probable prodromal DLB should be based on criteria for one of the three possible phenotypical presentations (MCI type, delirium type, or psychiatric type) accompanied by two core clinical features, among parkinsonism, RBD, visual hallucinations, fluctuation of attention, or one clinical feature and one proposed biomarker supportive of Lewy bodies pathology (14).

Most studies on prodromal DLB are of retrospective nature on MCI patients who had eventually developed DLB according to standard criteria. So far the MCI phenotype of DLB has been the one better characterized. In MCI-LB the type of cognitive impairment is usually non-amnestic, often multidomain and frequently associated with early visual and attentional deficits (27). In a prospective cohort study on MCI, the density estimate of the annualized competing risk of transitioning from non-amnestic MCI to probable DLB was 20%, against a 17% of amnestic MCI converting to probable AD (27). Conversion to DLB in patients with non-amnestic MCI may occur even more frequently than conversion to AD in patients with amnestic MCI (28).

Recently, Blanc and colleagues showed that in a group of prodromal DLB the cognitive profile was for most patients multidomain, including deficits in attention, visuoperceptual and visuospatial abilities. Performance on the Trail Making Test A, and on the fragmented letters and position discrimination subtests of the VOSP battery was more severely impaired in the prodromal DLB group than in a group of isolated subjective cognitive impairment and MCI without any core clinical feature of DLB (29).

Other authors assessed cognitive decline longitudinally and evaluated predictors of time to dementia in a group of patients with MCI-LB and in one with MCI-AD (30). At baseline, the MCI-LB group performed worse on executive and visuospatial tasks when compared with the MCI-AD group and, over time, the MCI-LB patients had a faster decline of attention. In contrast, the MCI-AD patients had lower baseline memory scores and showed a steeper decline of memory function over time when compared with the MCI-LB patients.

**Figure 1:**
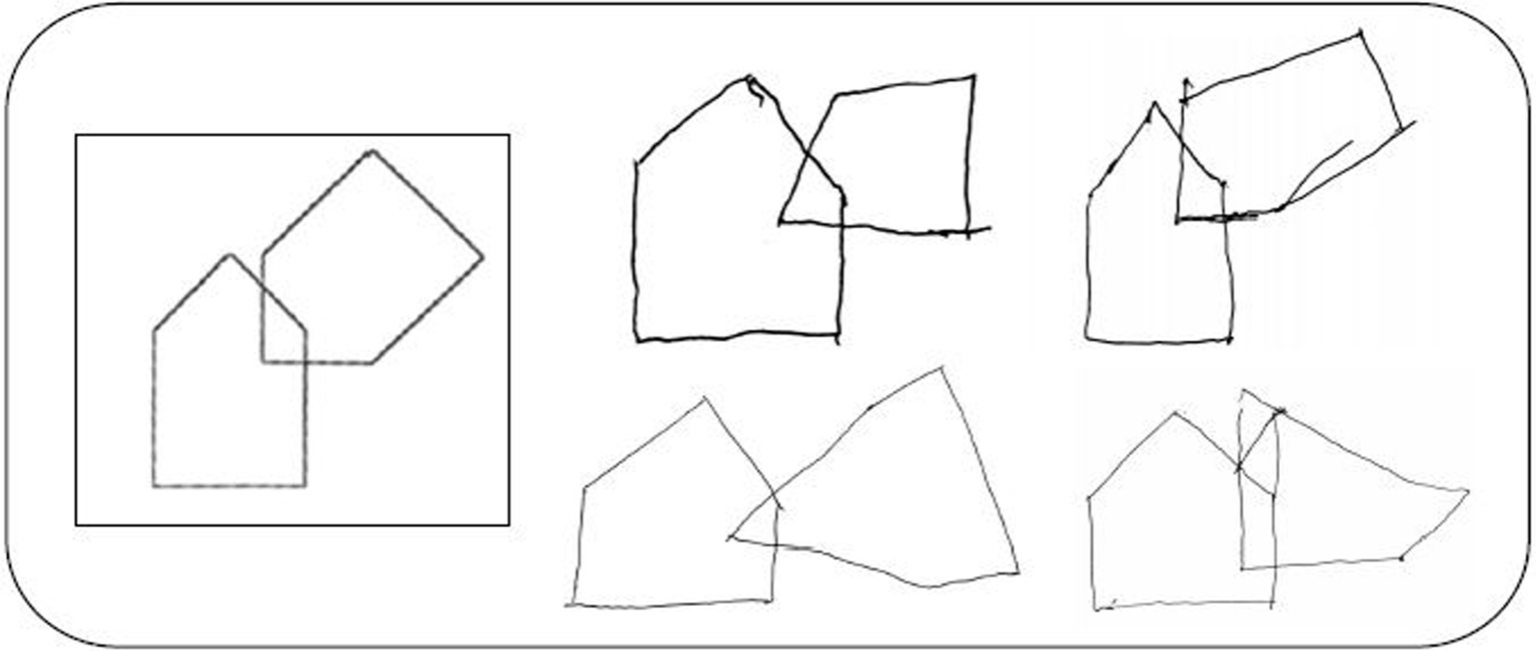
Examples of copies of the intersecting pentagons item of the MMSE by patients with MCI-LB.

The study by Cagnin and colleagues evaluated whether the use of a simple tool as the QSPT of the MMSE intersecting pentagon item could inform on the impaired visuoconstructional abilities in MCI-LB (mean MMSE score=28/30) (31). MCI-LB patients showed an impaired number of angles on the pentagon copy item more frequently than MCI-AD (MCI-LB: 45.1% versus AD 8.3%, p =0.005). The sensitivity of the impaired performance on this QSPT item was 41.4% (95% CI: 23%-59%) with a specificity of 91% (95% CI: 79%-100%) for differential diagnosis between prodromal DLB and AD. The positive predictive value was 85.7% (95% CI: 67%-100%) and negative predictive value was 54% (95% CI: 38%-70%). **Figure 1** shows some examples of copy of the intersecting pentagon item by MCI-LB patients. The impaired reproduction of the correct number of angles may be the first alteration in the progressive erosion of the ability to copy the intersection of pentagons detected in DLB (16, 31). Moreover, patients in the MCI-LB group showed greater impairment in speed of processing during visual attention (Trail Making Test- A: p= 0.026), and visuoconstructional (clock drawing: p= 0.029) tasks (30).

In a different sample of MCI-LB patients, Bussè et al. explored which visual space and object perception abilities were mostly impaired in prodromal DLB by using the VOSP battery (32). Visuoconstructional and visual motor tracking deficits were more severe in MCI-LB patients, similarly to those found in overt DLB by Calderon (24), while visuoperceptual abilities were impaired also in MCI-AD, particularly when assessing specific VOSP subtests such as the fragmented letters, silhouettes, object decision and progressive silhouettes items. In summary, the ventral visual pathway (‘what stream’) appeared to be involved in the prodromal stages in both DLB and AD, while functional impairment of the dorsal pathway (‘where stream’) and visuoconstructional abilities were more specific of prodromal DLB (32).

For the visuospatial domain, MCI-LB patients (mean MMSE=27.4/30) scored worse than healthy controls in the “number location” subtest of the VOSP (p = 0.047) and less significantly in the copy of the Complex Rey Figure (33). The copy of a complex figure also was more impaired in MCI-LB patients than in MCI-AD in a study by Yoon et al. (34). These studies suggest that in the initials stages of disease the copy of a complex figure is more sensitive than other tasks for detecting visuospatial and constructional deficits in prodromal DLB rather than in AD while in the intermediate stages no differences were found (18, 19).

Similar results were found in our study assessing MCI-LB in which impairments in the “number location”, as well as “cube analysis” subtests of the VOSP, and a trend to impairment in the copy of the Complex Rey Figure were more severe in MCI-LB than in MCI-AD (32). Moreover, patients in the MCI-LB group also showed greater impairment in speed of visual attention (Trail Making Test A, p = 0.03), working memory (digit span backward, p = 0.001) and executive functions (verbal fluency, p = 0.002).

In the study by Petrova et al., patients with very mild DLB (mean MMSE= 25.7) were impaired respect to healthy subjects a in copying abilities (test of “copy designs”), in the intersecting pentagon item from the MMSE and in the clock drawing test (35).

Overall, the few available studies on cognition in prodromal DLB have revealed that patients with MCI-LB have more severe visuospatial deficits and less impaired memory storage abilities than MCI-AD (27, 32, 35, 36).

## Episodic memory in dementia with Lewy bodies

Several studies compared memory impairment in DLB and AD showing less severe episodic memory impairment in the early stages of DLB (2, 37, 38). While the amnestic syndrome in AD is associated with damage of the mesial-temporal cortical regions (39), the neuropathological changes in DLB are widespread in neocortical regions (40, 41) with severe frontostriatal pathology (42). Therefore, different components of memory processing are affected in DLB and in AD. Memory impairment in AD is due to defective storage processes (2, 37), while in DLB is mainly driven by an elaborative encoding/strategic retrieval deficit, with less severe impairment of retention (41).

Cognitive evaluation of patients with autopsy-confirmed DLB showed better retention and recognition in verbal memory tests with the free and cued paradigm than that of patients with autopsy-confirmed pure AD (2). On the contrary, AD patients exhibited a more severe learning deficit, rapid forgetting and severely impaired recognition memory, suggesting a primary impairment of storage of information (2).

**Figure 2:**
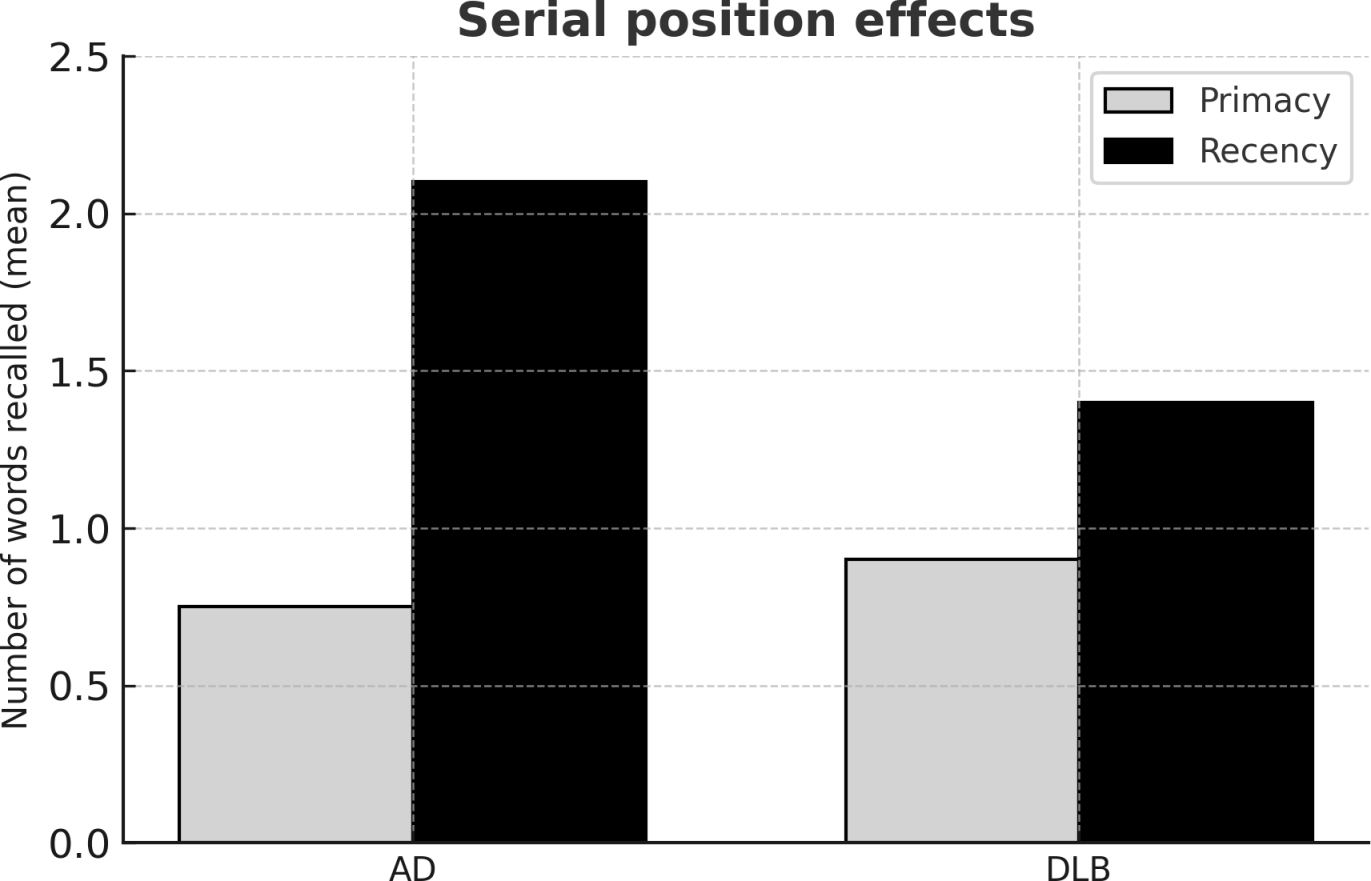
Mean values of number of words recalled in the primacy and recency segments of trial 1 of the Rey’s Auditory Verbal Learning Test. Histograms represent the mean values of the number of words recalled in the primacy (light grey) and recency (black) segments in dementia with Lewy bodies (DLB) and Alzheimer’s disease (AD) patients. In the primacy segment performance of DLB and AD patients was similarly different from that of healthy subjects (p<0.001); in the recency segment, performance of DLB patients was worse than that of AD patients (p<0.05).

Some authors have demonstrated that both DLB and AD (41, 43, 44) performed worse than healthy controls in the total score of the immediate and delay recall in word list tests. Considering the standard scores of the Rey Auditory Verbal Learning test (RAVLT), DLB patients did not perform differently from AD (19, 40, 45). In fact, global memory measures in standard tests, as the RAVLT, may not be sensitive enough to distinguish DLB from AD, while more detailed measures of verbal memory can offer more specific information. For example, patients with DLB may have a better learning curve, on the contrary, AD patients may have a more pronounced forgetting curve (19). The amnestic syndrome of AD, in fact, is characterized by deficient learning and rapid memory decay (39). Another interesting memory measure derives from the analysis of the serial position effect (46). Our group studied the recency effect with the RAVLT applied to DLB, AD and healthy participants (19). While global measures of immediate and delayed recall of RAVLT were similar in the two disease groups, DLB showed a reduction of the recency effect when compared with AD (**Figure 2**).

The Free and Cued Selective Reminding Test (FCSRT) is a valid tool to differentiate patients with AD from patients with non-AD dementias at a very mild stage (47) and to predict progression to AD in MCI individuals (48, 49). Therefore, the FSCRT could be more sensitive to detect an amnestic syndrome of the hippocampal type, based on significantly impaired performance in the cued recall with control of encoding test (50).

## Episodic memory in prodromal dementia with Lewy bodies

Early impairments in visual abilities and in the attentional domain have already been reported and, in MCI progressing to clinical probable DLB, attention and/or visuospatial impairments are concomitant with memory impairment (27, 28, 32).

The findings of a recent retrospective longitudinal study showed that MCI-LB performed better than MCI-AD on the delayed prose memory test (Wechsler Memory Scale), while the two groups were similarly impaired on the immediate recall part of this test (51).

A study by Bussè et al. showed that MCI-LB scored better than MCI-AD in selective measures of the FCSRT: immediate total recall (p = 0.01) and index of sensitivity of cueing (p = 0.001), while free delayed and total memory scores were similarly impaired. The index of sensitivity of cueing held a sensitivity of 76% and a specificity of 79% in distinguishing DLB (52).

Another study comparing the cognitive profile in MCI-LB used the French version of the FCSRT and found that patients with MCI-AD performed worse in both immediate and cued recall tasks (53).

In a cross-sectional study the authors investigated the profile of “very mild” (mean MMSE= 25.7/30) and “mild” (mean MMSE= 21.2/30) DLB patients and found that “very mild” DLB patients showed a memory profile typically described in subcortical dementia, including poor free recall, improved total recall, few intrusions, and relative sparing of recognition performance, suggesting a deficit of retrieval of information (35). In contrast, “mild” DLB patients had a memory profile that partially resembled a typical subcortical memory profile (impaired free recall with improved cued recall) but also that observed in cortical dementia (increased number of intrusions in immediate recall and impaired recognition (35, 54). These data suggest that the episodic memory impairment observed in very mild DLB is probably due to deficit of retrieval as well as encoding. These findings agree with an imaging study showing that hippocampal volumes and atrophy rates were within the normal range in the MCI-LB stage, but medial temporal atrophy increased with progression of dementia (55). When compared with healthy controls, 37 prodromal DLB patients performed worse on both free recall tasks (i.e., immediate and delayed) indicating weakened retrieval abilities (i.e., executive) rather than hippocampal verbal memory (i.e., storage) impairment (33). These findings are of importance because may highlight that verbal memory impairment can be present in DLB from its very early stages and it is not exclusively an indicator of prodromal AD. In a recent study, Querry and colleagues explored the memory profile of DLB, AD, and DLB/AD patients at a prodromal to mild stages in the context of a longitudinal follow-up over 48 months (56). These authors showed that DLB patients benefitted greatly from semantic cueing, their recognition and consolidation abilities were well-preserved, and both their verbal and visual memory performance remained remarkably stable over four years. Although at baseline evaluation there were not differences in visual memory between DLB and AD patients, either qualitatively or quantitatively, indicating that this test is less sensitive to detect performance differences between these two diseases, visual memory was stable over time in DLB, unlike AD and DLB/AD patients’ performances, which deteriorated significantly. Furthermore, authors highlight that a small number of these patients (2.3%) had a single total recall deficit, suggesting a potential impact of attentional fluctuations on memory abilities.

Regarding verbal memory, DLB patients had better performance compared to AD, showing a substantial benefit from semantic cueing. In accordance with previous studies, Querry and colleagues conclude that DLB patients present specific executive difficulties in retrieving information from memory rather than a storage deficit (56).

## Future research in context

In prodromal DLB, cognitive difficulties in visuospatial abilities and executive functions are prominent and are likely to account for cognitive disturbances observed in neuropsychological tests assessing other cognitive functions. Further studies should assess the relationship between memory, executive, attentional, and visuoperceptual performance, in order to clarify the primary and secondary contribution of visuospatial and executive deficits on cognitive performance in early stage DLB. In addition, the use of multimodal neuroimaging techniques could clarify the neural basis of these impairments.

Previous research has identified that DLB patients have a higher tendency to perceive a specific, often meaningful image in a random or ambiguous visual pattern, a phenomenon called pareidolia. Abnormal pareidolic responses are associated with severity of visual hallucinations (VH), and a pareidolia test can accurately classifies DLB with VH (57). However, Hamilton and colleagues showed that these findings are not confirmed at the prodromal DLB stage (i.e. MCI-LB) (58). Although, pareidolic responses are specifically more frequent in MCI-LB than MCI-AD, the relationship between hallucinations and pareidolic responses was not as clear as at the dementia stage, with comparisons limited by low rates of hallucinations in MCI (58). More recently, a study confirmed that performance at the Noise Pareidolia test is associated to performances in attention and visuospatial processing (59). The use of the Noise pareidolia test might be useful as a marker of risk of VH development associated with visual-perceptual deficits and over-interpretation. The accuracy of this test needs to be verified in cohorts of LBD patients with different disease severity but also with different phenotype at onset (i.e. psychiatric onset, delirium onset and motor onset).

Moreover, it has not yet established whether different phenotypes of onset are associated with profiles of cognitive functions differently affected.

We also envisage that application of computerized tools, particularly for the study of fluctuations of attentions and sustained attention, may be of utmost interest in the early LBD stage, with the advantage that these can be delivered through telemedicine techniques.

Finally, in this review we focused on the typical profile of cognitive impairment in LBD, i.e. visual-attentional deficits with relative sparing of verbal memory. We acknowledge that other domains not systematically addressed here, such as verbal fluency and sustained attention, may be of particular interest in the diagnostic work-up of LBD.

## Limitations of previous research

Unlike AD patients who mostly present an “hippocampal” memory profile characterized by deficits in encoding, storing, and consolidating information in both verbal and visual modalities, DLB patients benefit more from semantic cueing, showing good recognition and consolidation abilities (52, 56). The co-occurrence of AD and DLB is frequent and complicates individual patients’ diagnosis and management as well as the identification of the specific cognitive signs for each disease (13, 60). One of the main limitations of the published studies is the inclusion of both ‘pure DLB’ and DLB with mixed pathology in the same samples, therefore the use of biomarkers to exclude the presence of AD pathology or any other concomitant disease will be essential to characterize more in detail impairments specific to DLB. Another limitation of the available studies is that fluctuations, one of the core features of DLB, may have an impact on cognitive performance; therefore, especially in longitudinal studies, attentional fluctuations should be taken into account.

## Conclusions

The identification of the cognitive phenotype associated with early stage LBD is useful in the differential diagnosis among early neurodegenerative diseases and it is a long-standing and still actual topic. To this aim, neuropsychological research is continuously refining the tools to define more in detail the patterns of relatively preserved and impaired cognitive abilities that help differential diagnosis between DLB and AD at an early stage. A greater understanding of the cognitive distinctions between these disorders improves the ability to detect DLB patients at an early stage of disease providing an opportunity for early intervention.

This review provides evidence that a cognitive profile characterized by substantial impairments in visual cognitive abilities and relatively better performance on memory tasks that depend on hippocampal function characterizes the prodromal stage of DLB (26, 32, 33, 35, 52, 53).

In detail, testing of visuoconstructional abilities and usage of specific measures of memory performance that can differentiate between a deficit of storage and one of retrieval might be valuable for intercepting prodromal DLB. These cognitive measures could be incorporated in a screening battery and used together with clinical features predictive of synucleinopathies such as the presence of REM sleep behavior disorders and subtle extrapyramidal signs.

With progression of disease, and possibly presence of AD-related co-pathology, neuropsychological measures of visuospatial, executive and memory functions lose specificity and the neuropsychological profiles of DLB and AD are more similarly impaired, making differentiation of these two neurodegenerative diseases based only on neuropsychological measures less likely.
